# Dynamic Aha1 co‐chaperone binding to human Hsp90

**DOI:** 10.1002/pro.3678

**Published:** 2019-08-06

**Authors:** Javier Oroz, Laura J. Blair, Markus Zweckstetter

**Affiliations:** ^1^ Deutsches Zentrum für Neurodegenerative Erkrankungen (DZNE) Göttingen Germany; ^2^ Instituto de Química‐Física Rocasolano (IQFR‐CSIC) Madrid Spain; ^3^ Department of Molecular Medicine Morsani College of Medicine, USF Health Byrd Alzheimer's Institute, University of South Florida Tampa Florida; ^4^ Department of NMR‐based Structural Biology Max Planck Institute for Biophysical Chemistry Göttingen Germany

**Keywords:** Aha1, allostery, co‐chaperone, Hsp90, structure

## Abstract

Hsp90 is an essential chaperone that requires large allosteric changes to determine its ATPase activity and client binding. The co‐chaperone Aha1, which is the major ATPase stimulator in eukaryotes, is important for regulation of Hsp90's allosteric timing. Little is known, however, about the structure of the Hsp90/Aha1 complex. Here, we characterize the solution structure of unmodified human Hsp90/Aha1 complex using NMR spectroscopy. We show that the 214‐kDa complex forms by a two‐step binding mechanism and adopts multiple conformations in the absence of nucleotide. Aha1 induces structural changes near Hsp90's nucleotide‐binding site, providing a basis for its ATPase‐enhancing activity. Our data reveal important aspects of this pivotal chaperone/co‐chaperone interaction and emphasize the relevance of characterizing dynamic chaperone structures in solution.

**Abbreviations**
ADP: adenosine diphosphate
Aha1: activator of Hsp90 ATPase activity 1
AMP.PNP: 5′‐adenylyl‐β‐γ‐imidodiphosphate
ATP: adenosine‐5′‐triphosphate
FKBP51: FK506‐binding protein of 51 kDa
Hsp90: heat shock protein of 90 kDa
NMR: nuclear magnetic resonance
SAXS: small‐angle X‐ray scattering
TROSY: transverse relaxation‐optimized spectroscopy

## 1 INTRODUCTION

Heat shock protein of 90 kDa (Hsp90) is a highly conserved ATP‐dependent molecular chaperone responsible for the stabilization, maturation, and activation of many client proteins.^1^, ^2^ Several co‐chaperones regulate Hsp90's activation cycle, which is essential to maintain protein homeostasis.^3^, ^4^ During its activation cycle, the Hsp90 dimer undergoes large conformational rearrangements from an extended to a closed, ATPase‐active conformation, a process which involves intra‐ and inter‐protomer interactions.^5^, ^6^, ^7^ Different Hsp90 orthologs show distinct conformational equilibria^8^ that are translated into different ATPase activities. Human Hsp90 predominantly populates extended, ATPase‐incompetent dimeric conformations^7^, ^8^ and, compared to other orthologs, contains a very low inherent ATPase activity,^9^ despite the crucial role of Hsp90 ATPase activity for cell viability.^10^

Aha1 (*Activator of Hsp90 ATPase activity* 1)^11^ binds with an affinity of 0.5 μM to human Hsp90.^12^ The Aha1 co‐chaperone is unique in its ability to strongly enhance the inherently low ATPase activity of human Hsp90 and, thus, plays an important role as the facilitator for human Hsp90 to fulfill its activation cycle.^13^ Specifically, the Hsp90 dimer must close and both N‐terminal ATPase domains (Hsp90N) reposition to a dimerized state for ATP hydrolysis.^4^ Several intermediate steps in the closure process of Hsp90 were determined.^4^ Aha1 specifically helps to overcome the rate‐limiting conformational changes in Hsp90, which leads to a potent stimulation of its ATPase activity.^4^, ^9^, ^13^ However, the structural basis for this stimulation remains unknown.

Hsp90 is a very dynamic molecule,^5^ and its N‐terminal ATPase domain can freely rotate in solution.^14^ Aha1 was suggested to bind asymmetrically to the Hsp90 dimer and thus stabilize the interaction between the two Hsp90 N‐terminal domains in the closed dimer structure,^15^ in a nucleotide‐independent manner.^16^ Such a static model, however, is difficult to reconcile with the *cis* (Aha1 is bound to one protomer of the Hsp90 dimer) and *trans* (Aha1 is bound to both protomers of the dimer) interactions observed between Aha1 and Hsp90,^15^ especially considering the high degree of rotational freedom within Hsp90's N‐terminal domains.^14^ In addition, Aha1 was reported to promote a partially and not fully closed Hsp90 dimer conformation.^13^, ^15^ Only in the presence of nucleotide, Aha1 stabilizes the N‐terminally dimerized state of Hsp90 impeding the rotation of these domains.^14^ These observations suggest that the interaction between Hsp90 and Aha1 is complex and probably involves multiple transition states promoted by Aha1‐ and nucleotide‐binding, as well as ATP hydrolysis.^16^, ^17^ Here, using full‐length proteins, we reveal that the Hsp90/Aha1 complex is highly polymorph in the absence of nucleotide. Comparative analysis of perturbations observed in nuclear magnetic resonance (NMR) spectra indicate that Aha1 promotes conformational rearrangements in Hsp90 that favor ATP binding and hydrolysis.

## 2 RESULTS AND DISCUSSION

**Figure 1:**
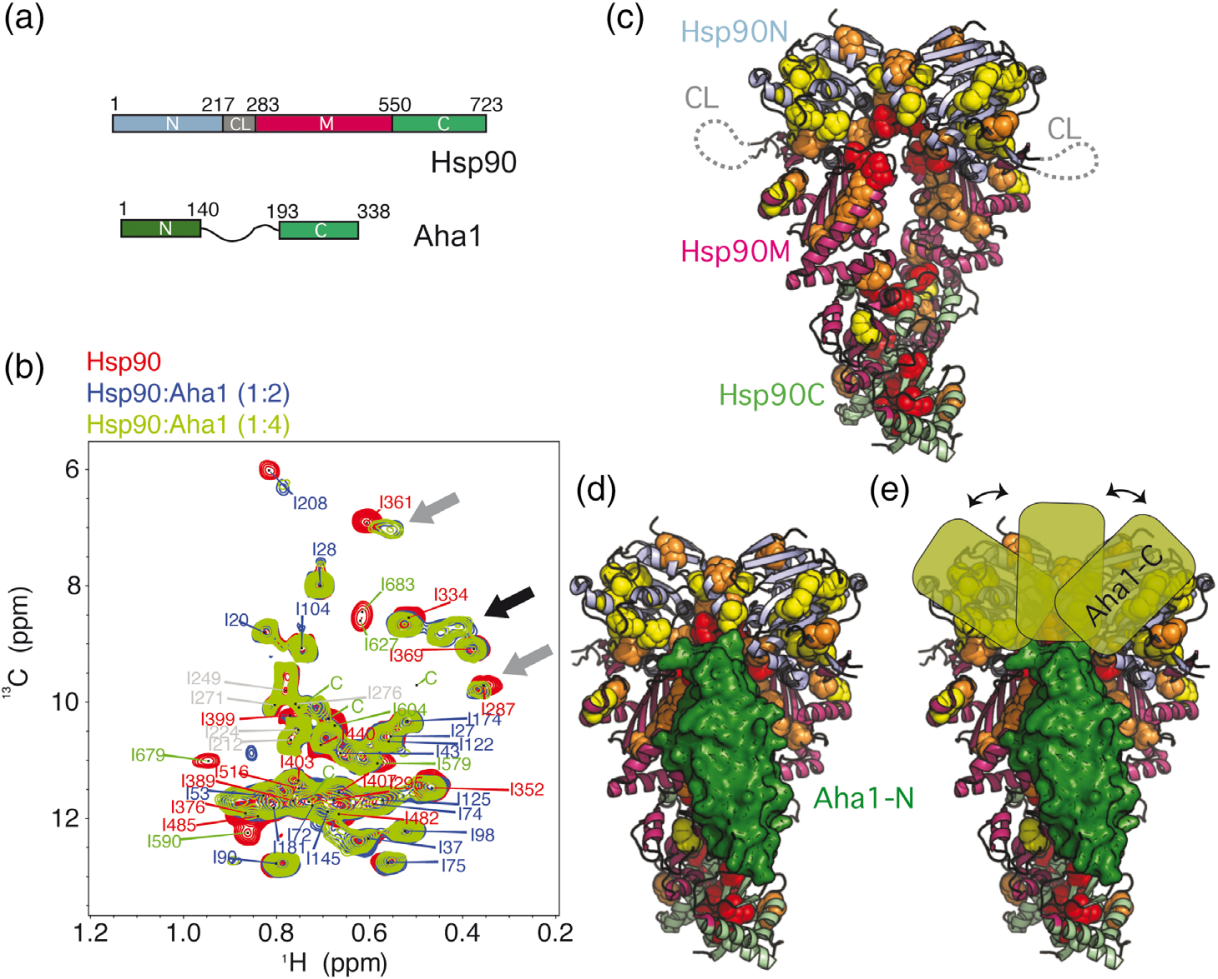
Interaction of Aha1 with human Hsp90 in the absence of nucleotide. (a) Domain organization of human Hsp90β and Aha1. The N‐terminal domain (Hsp90N) contains the ATPase active site, and Hsp90C is responsible for dimerization. The domain color code is kept in all figures. (b) Aha1 binding promotes strong changes in the methyl‐TROSY NMR spectra of Hsp90, including chemical shift perturbations and line broadening of Hsp90 isoleucine moieties (gray arrows) and the appearance of new signals (black arrow). The sequence‐specific assignment of cross‐peaks is indicated (Table S1). Four unassigned Hsp90C cross‐peaks are labeled with “c.” (c) Distribution of broadened Hsp90 isoleucine residues in Aha1 interaction and allostery on the structure of the closed Hsp90 dimer (PDB id 5fwk).^19^ Red, orange, and yellow spheres represent NMR perturbations of decreasing magnitude (shown in Figure S1b). The flexible charged linker of Hsp90 is represented by a dotted line. (d) Location of Aha1‐N (in green surface representation) on the structure of Hsp90 (PDB id 1usu).^20^ (e) The Aha1‐affected regions of Hsp90N suggest multiple bound conformations of the C‐terminal domain of Aha1 (Aha1‐C)

To gain insight into the structural basis of the allosteric binding of Aha1 to Hsp90, we studied the 214 kDa Hsp90/Aha1‐complex by solution NMR spectroscopy using methyl‐labeled human Hsp90β. Relaxation‐optimized NMR spectroscopy in combination with selective labeling has previously been used successfully to characterize dynamic, multi‐step interactions between Hsp90 and co‐chaperones and/or clients.^7^, ^18^ Addition of Aha1 to full‐length Hsp90 induced strong changes in the Hsp90 isoleucine NMR spectra (Figure 1a,b), including chemical shift perturbations and cross‐peak broadening, but also the appearance of new Hsp90 cross‐peaks (Figure 1b and Figure S1). The spectral changes indicate that, in the absence of nucleotide, Aha1 binds strongly to Hsp90 and promotes conformational rearrangements in the Hsp90 dimer, which shifts from a fully extended to a partially closed conformation.^7^, ^18^

Mapping of the affected isoleucines on the structure of Hsp90^19^ showed that the most strongly broadened residues are located at the interface between the middle (Hsp90M) and C‐terminal domain (Hsp90C) of Hsp90, as well as the N‐terminal interface involved in dimer closure (Figure 1c and Figure S1b). Both interfaces undergo rearrangements during Hsp90 allosteric changes.^20^ In addition, the regions affected in Hsp90M agree with the binding site of the N‐terminal domain of Aha1 (Aha1‐N), as displayed in the crystal structure of the complex of yeast Aha1‐N with Hsp90M (Figure 1c,d).^21^ Therefore, changes in Hsp90 NMR spectra in the presence of Aha1 reflect both the interaction and allosteric changes. Hsp90N, however, which would interact with Aha1‐C assuming a head‐to‐tail mode of interaction,^21^ was more broadly affected (Figure 1c,d and Figure S2), in agreement with footprinting of the Hsp90/Aha1‐interaction using crosslinking coupled to mass spectrometry,^12^ but contrary to a previously proposed model built on the basis of individual Hsp90 and Aha1 domain constructions.^15^ Because one Aha1 molecule binds to one Hsp90 dimer,^15^ the broad distribution of chemical shift perturbations in Hsp90N suggests that Aha1‐C binds in *cis* and *trans* 15 to multiple sites of both Hsp90N domains of the dimer leading to different Hsp90/Aha1‐complex structures (Figure 1e). The dynamic nature of the interaction may be supported by the structural flexibility that Hsp90N retains in complex with Aha1 in the absence of nucleotide^14^ and could further be favored by the flexibility of the long interdomain linker in Aha1 (Figure 1a). Notably, the interdomain linker in Aha1 is positively charged (pI = 8.3), whereas the flexible linker (CL)^22^ between Hsp90N and Hsp90M is negatively charged (Figure 1a,c; pI = 4.5), favoring electrostatic interactions between these parts of Aha1 and Hsp90. In addition to the polymorphic interactions on Hsp90N (Figure 1), Aha1 binding can induce conformational rearrangements on Hsp90N involving distant regions of the domain (Figures S2 and S3).

**Figure 2:**
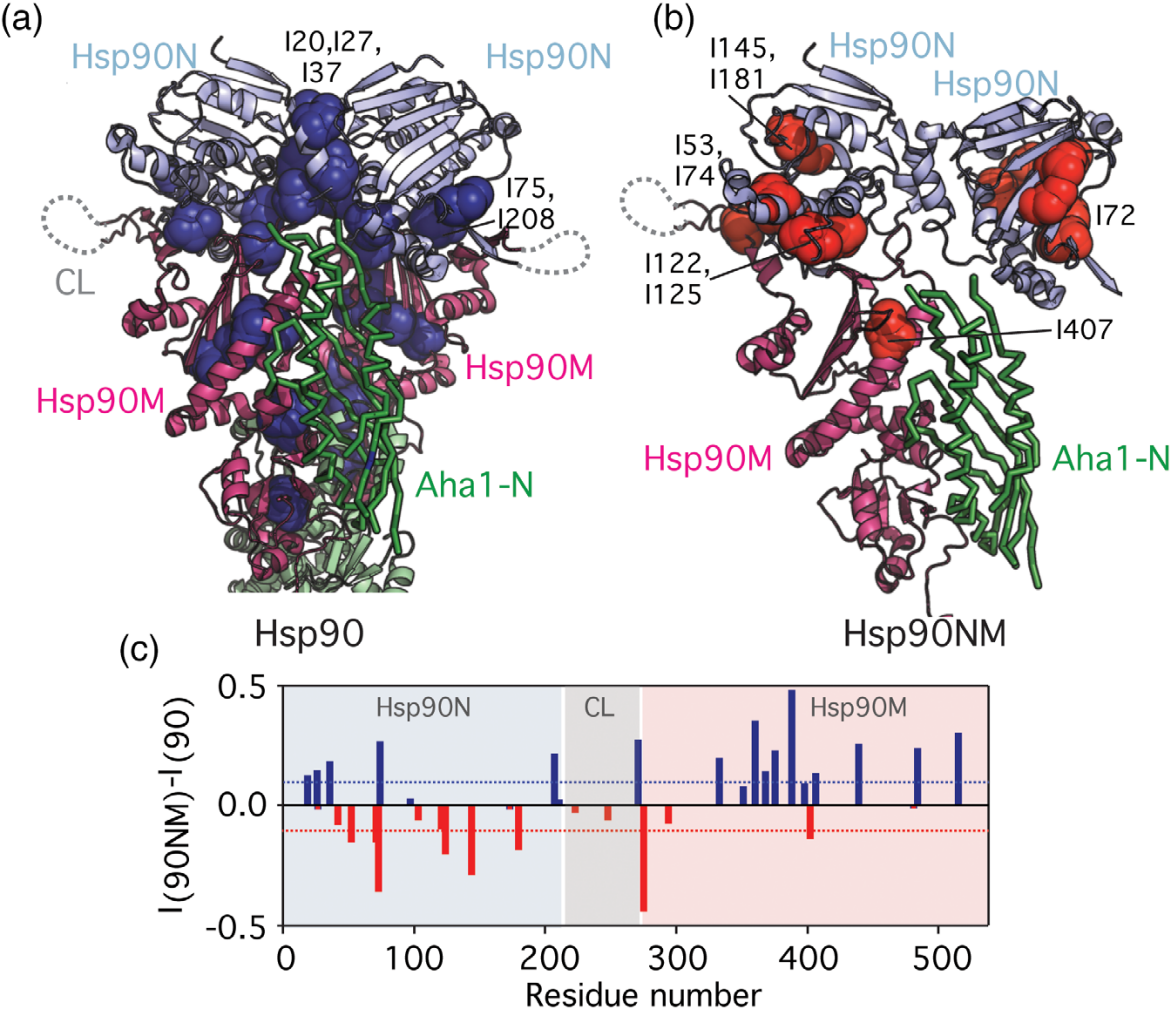
Discrimination between allosteric changes (a) and *cis* binding (b). (a) Mapping of residues that are more affected in the Hsp90/Aha1‐complex compared to the Hsp90NM/Aha1‐interaction (dark blue spheres, blue bars in c) on the structure of the closed Hsp90 dimer. (b) Residues that are more affected in the Hsp90NM/Aha1‐interaction when compared to the Hsp90/Aha1‐complex (red bars in c) are displayed with red spheres on Hsp90NM. To allow better comparison with panel (a), a second Hsp90N domain is displayed (as observed in the closed Hsp90 dimer; Figure 1c), as well as the N‐terminal domain of Aha1 (green). Labeled residues are mentioned in the text. (c) Comparison of Hsp90/Aha1‐ with Hsp90NM/Aha1‐interaction. (*I*/*I*_0_) ratios of Hsp90 isoleucine residues upon Aha1 interaction (Figure S1b) were subtracted from (*I*/*I*_0_) values observed for the Hsp90NM/Aha1‐interaction (Figure S4c; both at a molar ratio of 1:4). Positive values (blue bars) indicate isoleucine residues, which were more strongly broadened in full‐length Hsp90 upon addition of Aha1 (Hsp90/Aha1‐interaction), whereas negative values (red bars) indicate residues that were more strongly attenuated in Hsp90NM upon addition of Aha1 (Hsp90NM/Aha1‐interaction). Blue and red bars correspond to highlighted residues in (a, b)

**Figure 3:**
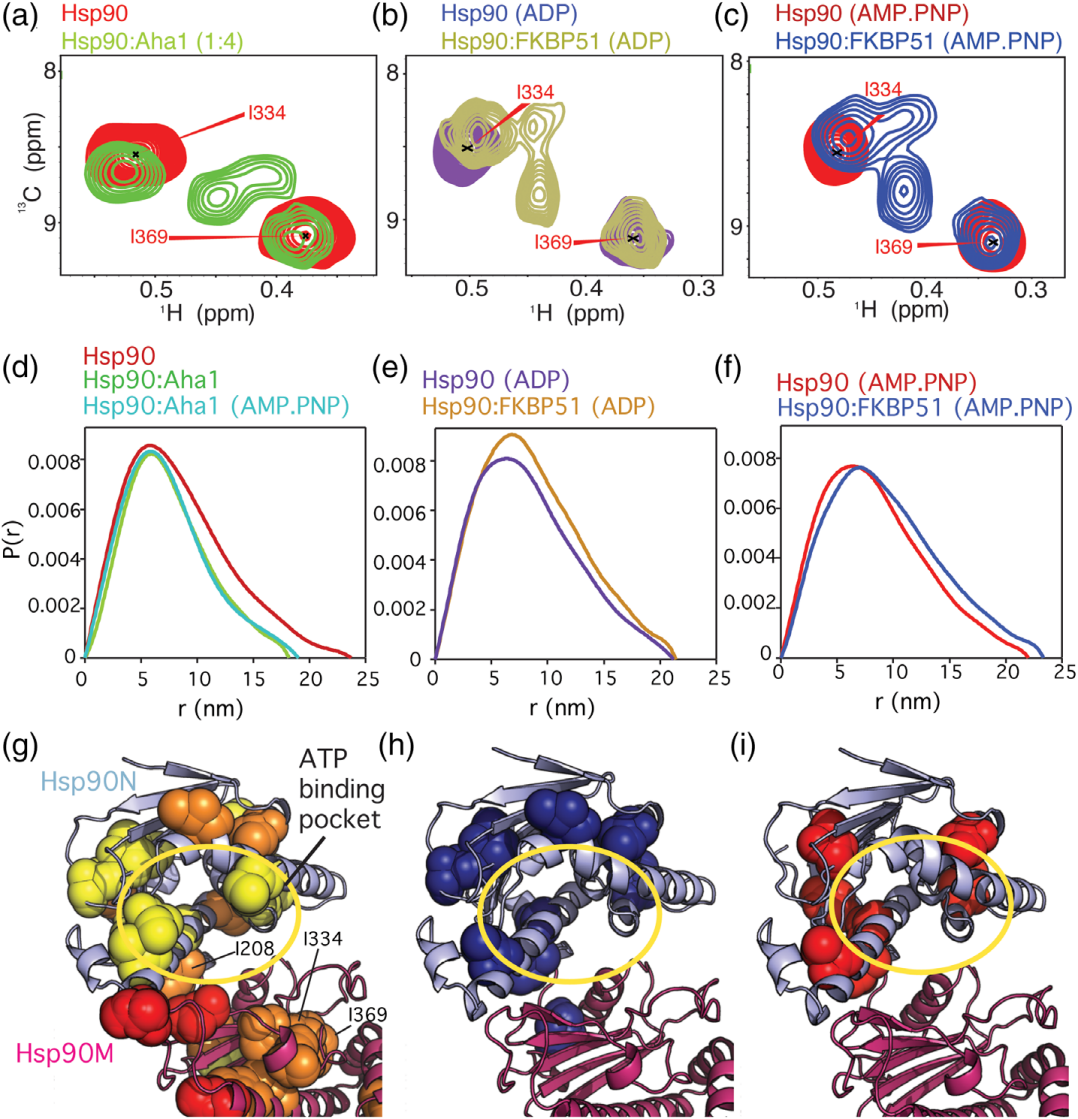
Structural changes induced by Aha1 and nucleotide binding. (a) New signals appear upon Aha1‐binding in the methyl‐TROSY spectra of full‐length Hsp90 in the absence of nucleotide. (b, c) New cross peaks appear upon binding of nucleotide and FKBP51. These signals are similar for ADP and AMP.PNP but are partially different from the peaks appearing in the presence of Aha1 (a). (d) SAXS *P*(*r*) distribution shows that Aha1 promotes a partially closed conformation^13^, ^15^ of Hsp90 independent of nucleotide. (e, f) FKBP51 stabilizes the extended conformation of Hsp90.^7^ (g) Aha1 promotes structural changes around the ATP‐binding pocket in Hsp90N in the absence of nucleotide. (h, i) Nucleotide binding promotes structural rearrangements around the ATP‐binding pocket.^7^ Spheres in (g–i) represent isoleucine residues affected in the corresponding binding process. A yellow circle in (g–i) highlights the ATP‐binding pocket

To discriminate between the binding of Aha1 and subsequent allosteric rearrangements induced in Hsp90 by the co‐chaperone, we prepared an Hsp90 construct containing only the N‐terminal and middle domains of Hsp90 (Hsp90NM), that is, the Hsp90 regions that are most relevant for the interaction. Because Hsp90NM is monomeric in solution,^7^ it cannot undergo the allosteric changes involved in dimer closure, but still allows the rotational freedom of Hsp90N.^14^ In addition, because Hsp90NM is unable to form a closed dimer, Aha1 will only be able to form *cis* interactions with Hsp90NM (Figure 2a,b). NMR data of the Hsp90NM/Aha1 interaction show that Aha1‐binding, although affecting similar regions on the chaperone (Figure S4a,b,d), was strongly attenuated when compared to full‐length Hsp90 (Figures S1b and S4c). Small‐angle X‐ray scattering (SAXS) of an equimolar Hsp90NM/Aha1‐mixture also resembled the scattering profile expected for a mixture of isolated proteins rather than for a stable complex (Figure S4e). The Hsp90NM/Aha1‐interaction is thus weak, in agreement with previous reports suggesting that the stable Aha1/Hsp90 interaction requires the presence of the Hsp90 dimer.^15^ The combined data show that interactions with both Hsp90 protomers are important for Aha1 stabilization,^12^ although Aha1 bound in *cis* to the full‐length Hsp90 dimer might reach the same level of ATPase stimulation.^15^ Overall, the data indicate that Aha1‐N establishes interactions with both Hsp90M domains upon induction of a partially closed conformation of the Hsp90 dimer (Figures 1c,(d), 2a, and 3d), whereas Aha1‐C forms transient interactions with both Hsp90N domains arranged in *cis* and *trans* in the Hsp90 dimer (Figure 1e).

Comparison of the perturbations induced by Aha1 in Hsp90 with those induced in Hsp90NM (Figure 2c) provide the unique opportunity to distinguish between Hsp90 regions affected only in the *cis* binding process (observed in Hsp90NM, Figure 2b) and those experiencing allosteric changes in the complex upon dimer closure (only observed in Hsp90, Figure 2a). Aha1 binding induces stronger effects on Hsp90M moieties for full‐length Hsp90 (Figure 2c), supporting that reduced Aha1‐N/Hsp90M interaction is responsible for the weaker binding of Aha1 to Hsp90NM. Remarkably, Hsp90N shows two clear interfaces: residues I20, I27, I37, I75, and I208 are more affected in the Hsp90/Aha1‐complex (Figure 2a,c). These residues are located in the Hsp90N dimer interface and in the Hsp90N/M interface, and thus the observed changes reflect allosteric rearrangements. However, I53, I72, I74, I122, I125, I145, and I181 were more strongly broadened in the Hsp90NM/Aha1‐interaction and form a continuous region on Hsp90N opposite to the dimer interface (Figure 2b,c), supporting the polymorphic interaction between the C‐terminal domain of Aha1 and the N‐terminal domain of Hsp90 (Figure 1e). Notably, only the Hsp90N dimer interface (Figure 2a) was proposed as potential Aha1‐C binding site in a previous model^15^ (Figure S2). Because Hsp90N can freely rotate even in complex with Aha1,^14^ the *cis* binding interface shown in Figure 2b could be rotated 180° upon Aha1‐binding.

To confirm the importance of the interaction of the N‐terminal domain of Aha1 with the middle domains of Hsp90, as seen in the complex structure of the two isolated domains^20^ and in our NMR data (Figure 2c), we characterized the binding of the mutant protein E67K‐Aha1 to Hsp90. E67K‐Aha1 is unable to stimulate the ATPase activity of Hsp90 due to impaired binding^12^ and failure to induce Hsp90 closed conformations and is ineffective in promoting Hsp90‐dependent maturation^12^ or aggregation of clients.^23^ Consistent with these data, binding of E67K‐Aha1 to human Hsp90 was weaker and did not result in new Hsp90 cross‐peaks (Figure S5a,b). Besides the mentioned need of both Hsp90M domains for a stable interaction with Aha1‐N, this impaired binding can be rationalized based on the atomic structure of the yeast Hsp90M/Aha1‐N‐complex.^20^ Residue E67 of Aha1 is surrounded in the complex by a cluster of positively charged residues of Hsp90M (Figure S5d,e) such that the E67K mutation results in electrostatic repulsion.

Binding of wild‐type Aha1 to full‐length Hsp90, but not to Hsp90NM, resulted in new Hsp90 cross‐peaks in the absence of nucleotide (Figure 1b and Figure S4a,b). The new Hsp90 signals could be due to inter‐protomer contacts established in the Hsp90N dimerization interface upon Aha1‐induced dimeric closure of Hsp90 (Figures 1c and 2a), or due to intra‐protomer conformational rearrangements in the chaperone induced by co‐chaperone action (Figure S3). To distinguish between these possibilities, we compared the Hsp90 spectrum in presence of Aha1 (without nucleotide, Figure 3a) with the Hsp90 spectrum when both the ATPase‐inhibiting co‐chaperone FKBP51^7^ and nucleotide were present (Figure 3b,c). In the latter conditions, the Hsp90 dimer will be extended, because FKBP51 impedes the allosteric closure of Hsp90 even in the presence of nucleotide (Figure 3e,f).^7^ The comparison showed that the new set of cross‐peaks, which appear upon Aha1‐binding in the absence of nucleotide (Figure 3a), partially resemble the new set of cross‐peaks that appear in the Hsp90/ADP/FKBP51 (Figure 3b) and Hsp90/AMP.PNP/FKBP51 complexes (Figure 3c). In agreement with previous results,^4^ SAXS analysis of the Hsp90/Aha1‐complex showed that co‐chaperone binding induced a partially closed conformation of Hsp90 in both the absence and presence of AMP.PNP (Figure 3d). The comparison suggests that the Aha1‐driven, complex‐specific Hsp90 signals arise from conformational changes induced in Hsp90 by Aha1‐binding, which are independent of inter‐protomer allosteric closure.

Binding of nucleotides to Hsp90 induces a rotation of Hsp90N^14^, ^24^ and structural changes in the ATP lid^5^, ^21^ (Figure S3a), which promote the trapping of the nucleotide in the binding pocket.^25^ The spectral changes of Hsp90 upon Aha1 binding in the absence of nucleotide are similar, but not identical to those induced by nucleotide binding (Figure 3a–c and Figure S3). This suggests that Aha1 induces intermediate conformational rearrangements around the ATP‐binding pocket of Hsp90N, which still allow for ATP exchange^13^ but that energetically facilitate the additional changes required for ATP trapping and hydrolysis.^26^, ^27^ Consistent with this hypothesis, NMR spectroscopy indicated that Aha1 promotes rearrangements on Hsp90N that resemble those induced by Hsp90N dimerization (Figure S3b), priming the domain for nucleotide trapping.

Our data support the multi‐step nature of the Hsp90/Aha1‐interaction,^17^ where the N‐terminal domain of Aha1 interacts with both Hsp90M domains upon induction of a partially closed conformation of the Hsp90 dimer. In addition, the data indicate that the C‐terminal domain of Aha1 can adopt several conformations leading to a dynamic, polymorphic complex. The N‐terminal domains of Hsp90, which remain flexible in the complex,^14^ undergo conformational rearrangements towards an intermediate state that facilitates ATP binding. Because Aha1 is critical for accelerating the ATPase activity of human Hsp90,^13^ our study helps in deciphering the molecular mechanism of this stimulation and, thus, in the understanding of the activation cycle of Hsp90, which is fundamental to maintain eukaryotic homeostasis.^3^, ^4^

## 3 MATERIALS AND METHODS

Human Hsp90β and Aha1 were cloned into pET28b and pET21a vectors, respectively (Novagen), and expressed in BL21(DE3) *E. coli* strain. Metabolic precursors for selective [^1^H‐^13^C]‐labeling of Hsp90 isoleucine δ_1_ methyl groups in fully deuterated media were purchased from NMR‐Bio. Selective labeling was achieved as described in Reference 28. Cells were lysed by sonication and recombinant proteins purified by Ni^2+^ affinity chromatography in Ni^2+^‐NTA agarose (Thermo Fisher) using 50 mM TrisHCl/500 mM NaCl/10 mM imidazole (pH 8.0) as binding buffer, increasing to 250 mM imidazole for elution. Aha1 (both *wt* and E67K variant) was subjected to tobacco etch virus (TEV) proteolysis and subsequently purified again by Ni^2+^ affinity purification. Proteins were further purified by size exclusion chromatography in 10 mM Hepes/500 mM KCl/1 mM DTT (pH 7.5) using a Superdex 200 column (GE Healthcare). Pure proteins were concentrated and stored at −80°C.

NMR experiments in the absence of nucleotide (Figures 1b and 3a; Figures S1, S4, and S5) were acquired at 25°C on Bruker Avance III 800 and 900 MHz spectrometers (both equipped with TCI cryoprobes) using 50 mM sodium phosphate/300 mM NaCl/1 mM DTT (pH 7.2) in 100% D_2_O. Spectra in the presence of nucleotide (1 mM ADP or AMP.PNP, Figure 3b,c) were obtained in 20 mM Hepes/5 mM KCl/10 mM MgCl_2_/1 mM DTT (pH 7.4) in 100% D_2_O. Of note, 80–100 μM isoleucine‐labeled Hsp90 or Hsp90NM were used for NMR titrations. NMR binding plots show the decay in signal intensity (Figures S1, S4, and S5), where *I*_0_ is the intensity of the cross‐peaks in the reference spectra. Assignment of Hsp90 isoleucine δ_1_ methyl groups is described in detail in Reference 7. Chemical shifts for Hsp90 isoleucine methyl groups (Figure 1b) are listed in Table S1. Spectra were processed using TopSpin (Bruker) and analyzed in Sparky (Goddard & Kneller, UCSF).

SAXS data were collected at 25°C from pure and monodisperse samples of Hsp90, Hsp90NM, and Aha1 in 50 mM sodium phosphate/10 mM NaCl/1 mM DTT (pH 6.8) (Figure 3d and Figure S4e) or 20 mM Hepes/5 mM KCl/10 mM MgCl_2_/1 mM DTT (pH 7.4) in the presence of 2 mM AMP.PNP (Figure 3d,f) or 2 mM ADP (Figure 3e). Sample concentrations ranged from 20 to 100 μM and shown *P*(*r*) distributions contained 50 μM Hsp90/Hsp90NM and equimolar amounts of Aha1. Scattering profiles were analyzed using standard procedures using ATSAS.^29^ A theoretical Hsp90NM/Aha1 complex curve was calculated using the program CRYSOL available in the ATSAS package (Figure S4e). In brief, the theoretical Hsp90NM/Aha1 complex was built using the size of the individual proteins as obtained by experimental SAXS (Figure S4e), which were forced to interact following a structural alignment with the available Hsp90M/Aha1‐N complex (PDB id 1usu).^20^ The resulting complex structure was submitted to CRYSOL for size determination. Data collection for the Hsp90/FKBP51/nucleotide complexes is described in Reference 7. SAXS measurements were performed at DESY (Hamburg, Germany) and Diamond Light Source (Oxford, UK) stations.

## AUTHOR CONTRIBUTIONS

J.O. performed the experiments and analyzed the data; J.O., L.J.B., and M.Z. conceived the project; J.O. and M.Z. wrote the manuscript.

## CONFLICT OF INTEREST

The authors declare no conflict of interest.

## Supporting information


**Appendix S1**: Supporting Information
