# Supplementary material for: Dynamic Aha1 co‐chaperone binding to human Hsp90
Source: Protein Sci. 2019 Aug 6;28(9):1545–51. doi: 10.1002/pro.3678 (PMC6699087; doi:10.1002/pro.3678)
Supplement: Supplementary file 1 — Appendix S1: Supporting Information [file PRO-28-1545-s001.pdf]

Supplementary Information for

Dynamic Aha1 Co-Chaperone Binding to Human Hsp90

Javier Oroz, Laura J. Blair, and Markus Zweckstetter

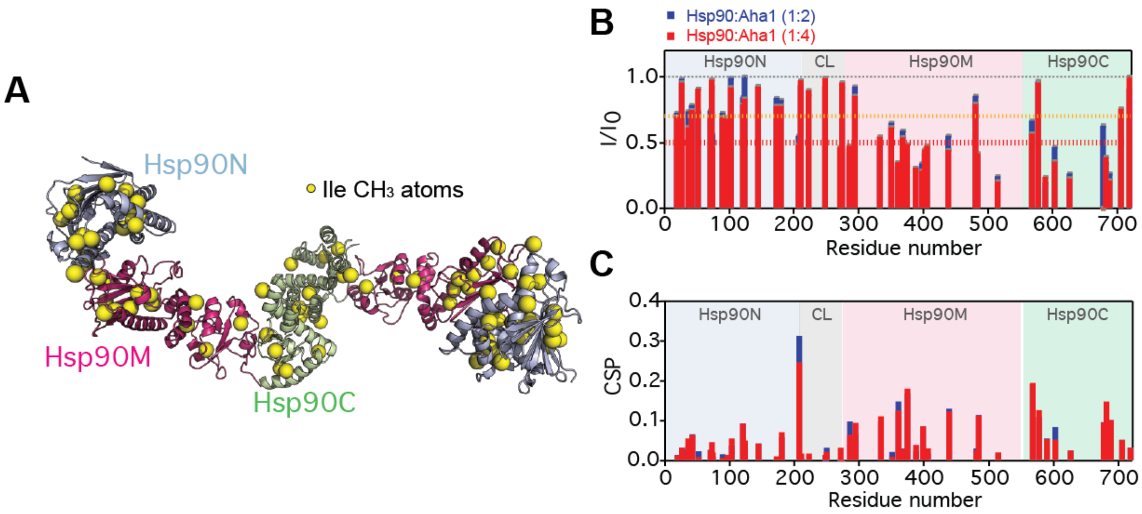

Supplementary Figure 1. NMR spectroscopy of the Hsp90/Aha1-interaction.
A) Localization of ^{13}C-labeled isoleucine methyl groups (yellow spheres) on the extended conformation of the human Hsp90 dimer (1) (modified from PDB id 5fwk) (2).
B) Sequence-specific analysis of the changes in Hsp90 peak intensity upon addition of Aha1. Blue and red bars represent 1:2 and 1:4 molar ratios, respectively. The orange and red lines mark I/I_0 thresholds of 0.7 and 0.5, respectively, to color code residues in Figure 1C-E: $I/I_0<0.5$ in red; I/I_0= 0.5-0.7 in orange; I/I_0= 0.7-1 in yellow. Error bars (in grey, very small) were calculated using the spectral signal-to-noise ratios.
C) Chemical shift perturbation plot of the Hsp90/Aha1-interaction. Hsp90 domains are indicated.

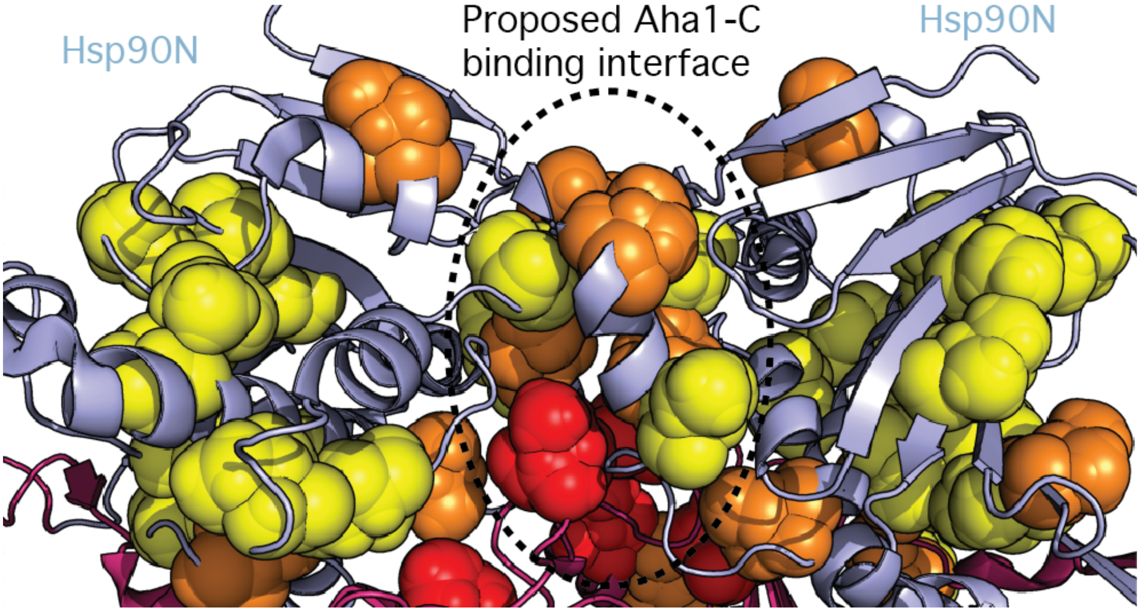

Supplementary Figure 2. Multiple regions of the N-terminal domain of Hsp90 are affected by Aha1 binding.

Isoleucine residues of the N-terminal domains of Hsp90, which are perturbed by addition of Aha1 to full-length Hsp90 (Figure 1B), are represented by spheres using the structure of the closed Hsp90 dimer (PDB id 5fwk). The color code is the same as used in Figures 1 and S1. The previously proposed Aha1-C binding interface (3) is marked.

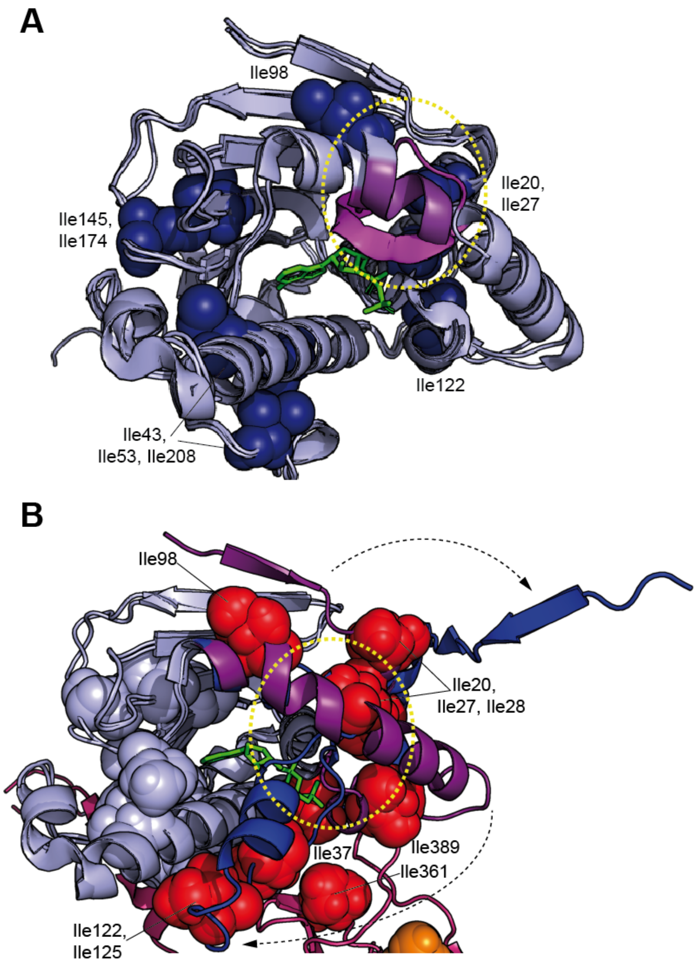

Supplementary Figure 3. Conformational changes in the N-terminal domain of Hsp90 upon nucleotide binding and dimerization.
A) Superposition of the structure of the N-terminal domain of Hsp90 (Hsp90N) in the absence of ADP (PDB id: 5j2v) with two structures in the presence of ADP (PDB id: 1am1, ADP-bound yeast Hsp90; 1uym, Hsp90Nb bound to the PU3 analog of ATP). ADP is displayed with green sticks. Nucleotide binding to Hsp90N induces changes in the proximal part of helix 4 (highlighted in magenta, yellow circle). Isoleucine residues, for which the methyl cross-peaks are perturbed by binding of ADP to full-length Hsp90 (please see Figure 3H), are displayed with blue spheres.
B) Superposition of monomeric Hsp90N structure (PDB id: 1uym) with Hsp90N in the Hsp90 dimer (PDB id: 5fwk), revealing large conformational changes (indicated by arrows) during nucleotide binding, dimer closure and Hsp90N dimerization: β-Strand 1 swaps between domains and helix 4 folds around the hinge (yellow circle) to fully close the nucleotide binding pocket. Regions undergoing strong conformational changes are marked in magenta and dark blue in monomeric and dimeric Hsp90N, respectively. Isoleucine residues, for which the methyl cross-peaks are perturbed upon addition of Aha1 to full-length Hsp90 in the absence of nucleotide (Figures 1, S1) are displayed by spheres (red and light blue). I20, I27, I28, I37, I98, I122 and I125 probe conformational rearrangements in Hsp90N upon dimerization, while I361 and I389 are located on the Hsp90M loop that clamps the new conformation of Hsp90N's helix 4. All these isoleucine residues are affected upon Aha1 binding in the absence of nucleotide, suggesting that the co-chaperone induces conformational rearrangements in Hsp90N similar to those induced by nucleotide binding and dimerization. Light blue spheres mark the Aha1 *cis* binding interface of Hsp90N (Figure 2B). ATP is displayed with green sticks.

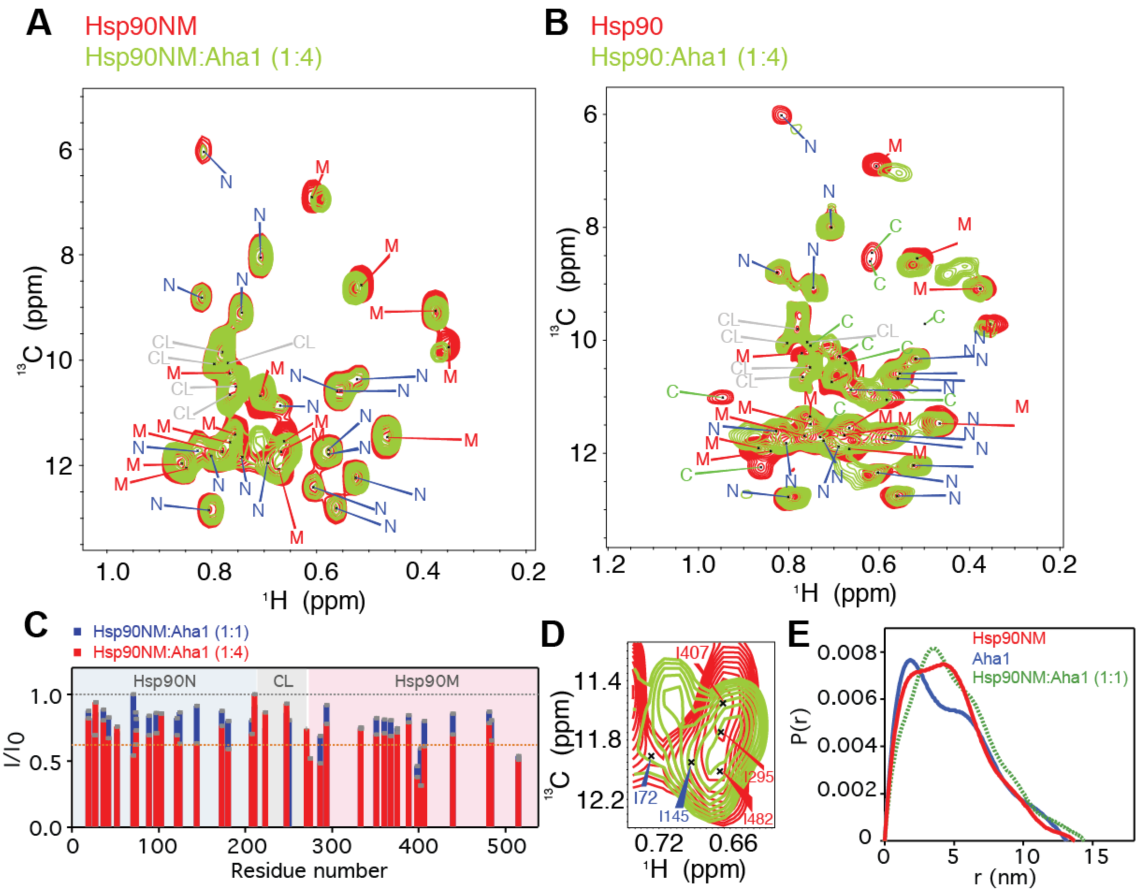

Supplementary Figure 4. Aha1 binds weakly to monomeric Hsp90NM.
A-B) Methyl-TROSY spectra of Hsp90NM showing that Aha1-binding does not promote the appearance of new isoleucine cross-peaks (A), in contrast to Aha1-binding to full-length, dimeric Hsp90 (B, same as in Figure 1B). Molar ratios are indicated. For clarity, cross-peaks were labeled relative to Hsp90 domains.
C) Sequence-specific decrease in the intensities of methyl cross-peaks of Hsp90NM upon addition of equimolar (blue) and 4-fold molar excess (red) of Aha1. An orange dashed line at I/I_0=0.7 marks the threshold below which Hsp90NM residues are considered to be significantly affected upon addition of Aha1. Error bars (grey bars, very small) are based on spectral S/N ratios.
D) Selected region of the Hsp90NM methyl-TROSY spectra in the absence (red) and presence (green) of Aha1 (as shown in panel (A)). I72 and I407 were strongly shifted.
E) SAXS P(r) distribution of the mixture (in green) indicates that the complex is not stable and that it is highly dynamic and/or polymorphic. A theoretical curve for the stable Hsp90NM/Aha1 complex is represented in black dotted line.

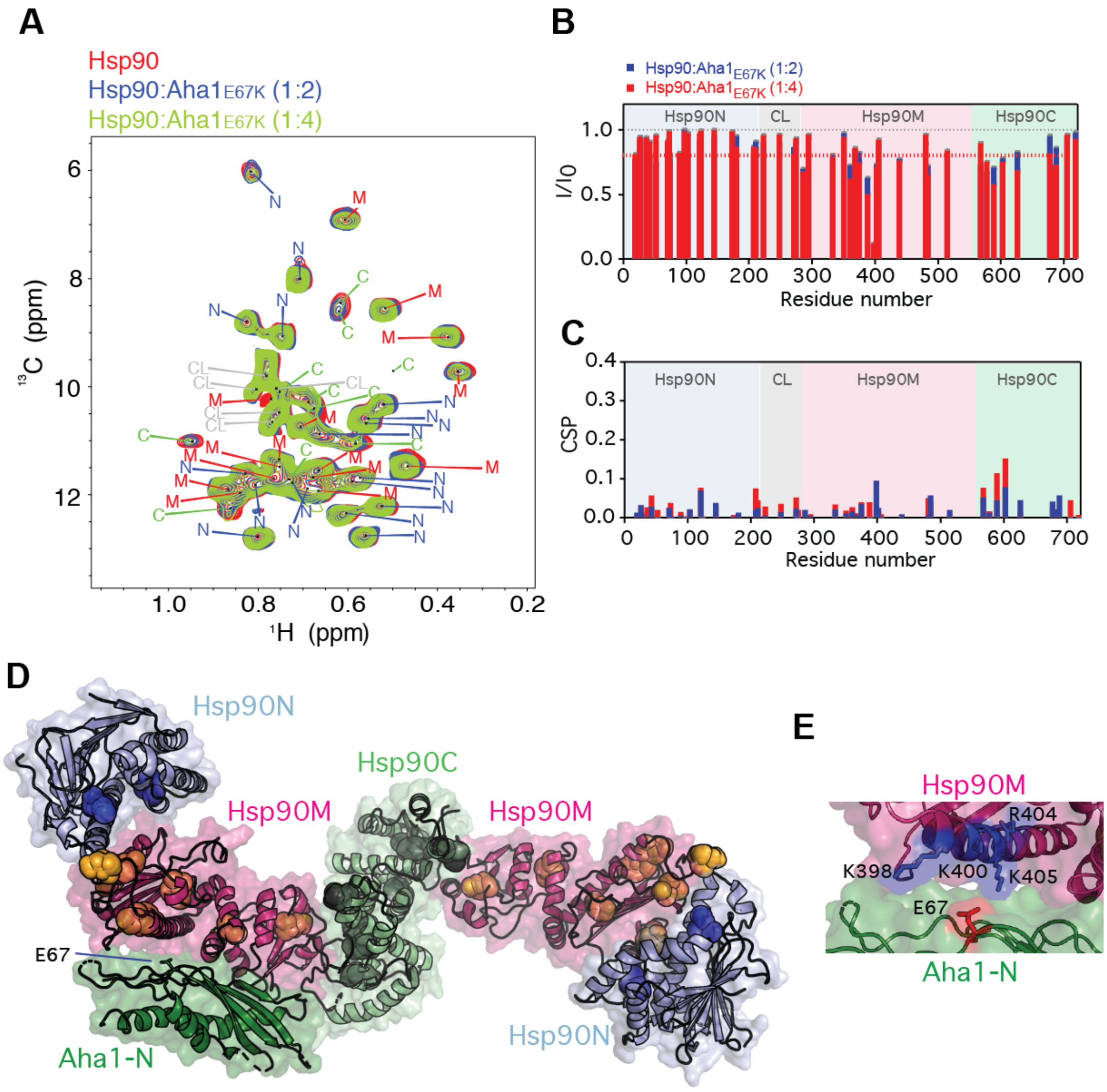

Supplementary Figure 5. E67K mutation in the N-terminal domain of Aha1 diminishes binding to human Hsp90.

A) Superposition of isoleucine methyl-TROSY spectra of Hsp90 in the absence (red) and presence of a 2-fold (blue) and 4-fold molar excess of the E67K-mutant Aha1. For clarity, cross-peaks were labeled relative to Hsp90 domains.

B-D) Sequence-specific changes in the intensity (B) and position (C) of isoleucine methyl cross-peaks of Hsp90 upon addition of the E67K-mutant Aha1 (as displayed in panel (A)). Error bars (in grey, very small) were calculated based on spectral S/N ratios. Isoleucine residues with intensity ratios (I/I_0) below 0.8 are displayed as spheres in (D), with the N-terminal domain of Aha1 (Aha1-N) positioned according to PDB id 1usu (4). The Hsp90 dimer is shown in an extended, ATP-incompetent conformation (modified from PDB id 5fwk (2)) following the known inability of the E67K-mutant Aha1 to stimulate Hsp90's ATPase activity (5).

E) The side-chain of E67 (in red sticks) in Aha1-N is surrounded by a cluster of positively charged residues of Hsp90M (K398, K400, R404 and K405, represented in blue sticks).

Supplementary Table 1. Hsp90 Ile δ^1 CH_3 assignments. Four unassigned residues in Hsp90C domain are marked with C.

Domain	Residue	^{13}C (ppm)	^{1}H (ppm)
Hsp90N	I20	8.802	0.826
	I27	10.587	0.554
	I28	8.001	0.706
	I37	12.343	0.603
	I43	10.878	0.663
	I53	11.607	0.828
	I72	11.779	0.723
	I74	11.752	0.59
	I75	12.756	0.56
	I90	12.773	0.801
	I98	12.214	0.524
	I104	9.069	0.746
	I122	10.678	0.559
	I125	11.686	0.573
	I145	11.882	0.69
	I174	10.327	0.519
	I181	11.82	0.806
	I208	6.019	0.816
	I212	10.647	0.769
CL	I224	10.478	0.753
	I249	9.799	0.781
	I271	10.048	0.804
	I276	10.032	0.759
Hsp90M	I287	9.717	0.355
	I295	11.679	0.677
	I334	8.549	0.516
	I352	11.474	0.466

	I361	6.915	0.606
	I369	9.086	0.376
	I376	11.907	0.867
	I389	11.693	0.765
	I399	10.229	0.771
	I403	11.347	0.753
	I407	11.553	0.666
	I440	10.737	0.705
	I482	11.923	0.666
	I485	11.962	0.84
	I516	11.483	0.752
Hsp90C	I579	11.053	0.583
	I590	12.247	0.862
	I604	10.403	0.675
	I627	8.603	0.62
	I679	11.014	0.946
	I683	8.448	0.616
	C	10.282	0.688
	C	9.709	0.499
	C	11.713	0.731
	C	10.093	0.752

References

1. Oroz J, Chang BJ, Wysoczanski P, Lee CT, Perez-Lara A, Chakraborty P, Hofele RV, Baker JD, Blair LJ, Biernat J, Urlaub H, Mandelkow E, Dickey CA, Zweckstetter M (2018) Structure and pro-toxic mechanism of the human Hsp90/PPIase/Tau complex. Nature Communications 9:4532.
2. Verba K, A., Wang RY-R, Arakawa A, Liu Y, Shirouzu M, Yokoyama S, Agard DA (2016) Atomic structure of Hsp90-Cdc37-Cdk4 reveals that Hsp90 traps and stabilizes an unfolded kinase. Science 352:1542-1547.

3. Retzlaff M, Hagn F, Mitschke L, Hessling M, Gugel F, Kessler H, Richter K, Buchner J (2010) Asymmetric activation of the Hsp90 dimer by its cochaperone Aha1. Molecular Cell 37:344-354.
4. Meyer P, Prodromou C, Liao C, Hu B, Roe SM, Vaughan CK, Vlasic I, Panaretou B, Piper PW, Pearl LH (2004) Structural basis for recruitment of the ATPase activator Aha1 to the Hsp90 chaperone machinery. EMBO Journal 23:1402-1410.
5. Koulov AK, LaPointe P, Lu B, Razvi A, Coppinger J, Dong M-Q, Matteson J, Laister R, Arrowsmith C, Yates III JR, Balch WE (2010) Biological and structural basis for Aha1 regultaion of Hsp90 ATPase activity in maintaining proteostasis in the human disease cystic fibrosis. Molecular Biology of the Cell 21:871-884.
